# Tumor treating fields suppress tumor cell growth and neurologic decline in models of spinal metastases

**DOI:** 10.1172/jci.insight.176962

**Published:** 2024-03-21

**Authors:** Daniel Ledbetter, Romulo Augusto Andrade de Almeida, Xizi Wu, Ariel Naveh, Chirag B. Patel, Queena Gonzalez, Thomas H. Beckham, Robert North, Laurence Rhines, Jing Li, Amol Ghia, David Aten, Claudio Tatsui, Christopher Alvarez-Breckenridge

**Affiliations:** 1 Department of Neurosurgery, University of Texas MD Anderson Cancer Center, Houston, Texas, USA.; 2 Novocure Ltd., Haifa, Israel.; 3 Department of Neuro-Oncology,; 4 Department of Radiation Oncology, CNS/Pediatrics Section, and; 5 Department of Diagnostic Imaging, University of Texas MD Anderson Cancer Center, Houston, Texas, USA.

**Keywords:** Bone biology, Neuroscience, Neurological disorders, Orthopedics

## Abstract

Spinal metastases can result in severe neurologic compromise and decreased overall survival. Despite treatment advances, local disease progression is frequent, highlighting the need for novel therapies. Tumor treating fields (TTFields) impair tumor cell replication and are influenced by properties of surrounding tissue. We hypothesized that bone’s dielectric properties will enhance TTFields-mediated suppression of tumor growth in spinal metastasis models. Computational modeling of TTFields intensity was performed following surgical resection of a spinal metastasis and demonstrated enhanced TTFields intensity within the resected vertebral body. Additionally, luciferase-tagged human KRIB osteosarcoma and A549 lung adenocarcinoma cell lines were cultured in demineralized bone grafts and exposed to TTFields. Following TTFields exposure, the bioluminescence imaging (BLI) signal decreased to 10%–80% of baseline, while control cultures displayed a 4.48- to 9.36-fold increase in signal. Lastly, TTFields were applied in an orthotopic murine model of spinal metastasis. After 21 days of treatment, control mice demonstrated a 5-fold increase in BLI signal compared with TTFields-treated mice. TTFields similarly prevented tumor invasion into the spinal canal and development of neurologic symptoms. Our data suggest that TTFields can be leveraged as a local therapy within minimally conductive bone of spinal metastases. This provides the groundwork for future studies investigating TTFields for patients with treatment-refractory spinal metastases.

## Abstract 2

Computational, in vitro, and in vivo models of spine metastases support the use of tumor-treating fields for treatment of these osseous tumors.

## Graphical abstract

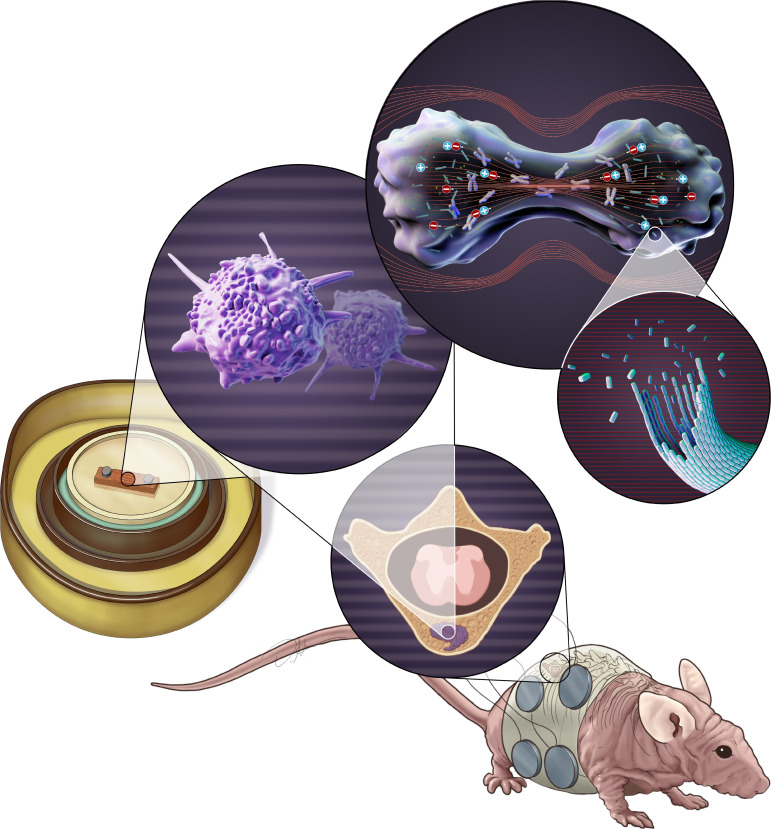

## Introduction

The management of spinal metastases typically requires the combination of local therapy and concurrent systemic treatment. Surgery followed by conventional external beam radiotherapy (cEBRT) (1) is the standard of care for symptomatic metastatic spinal cord compression. However, this paradigm has changed with the emergence of spinal stereotactic radiosurgery (SSRS). A common approach is to treat radiosensitive tumors (e.g., hematological malignancies, breast, and prostate cancer) with cEBRT, while radioresistant tumors (e.g., renal cell carcinoma, melanoma, non–small cell lung cancer, sarcoma, etc.) are treated with SSRS either alone (2–4) or in the postoperative setting (5–7).

The number of individuals diagnosed with spinal metastases is expected to increase as targeted agents (8, 9) and immune therapies (10) improve the overall survival of cancer patients. In this context, the ideal treatment of spinal tumors should not disrupt the management of systemic disease. While radiation therapy is effective in the majority of patients with spinal metastases, recurrence after cEBRT is common and some patients develop multiply recurrent tumors not amenable to further radiation therapy (11, 12). Although numerous therapeutic strategies for these patients have been described (13–17), these interventions are unlikely to prevent disease progression in radiation-refractory tumors.

Tumor treating fields (TTFields) is an emerging treatment modality that utilizes intermediate-frequency (100–500 kHz), low-intensity (1–3 V/cm) alternating electrical fields to disrupt cancer cell replication (18). TTFields is currently approved for the treatment of recurrent glioblastoma as monotherapy, newly diagnosed glioblastoma when combined with adjuvant temozolomide, and malignant pleural mesothelioma when combined with concomitant pemetrexed and platinum-based chemotherapy (19–22). This form of treatment is noninvasively delivered by cutaneous transducer arrays overlying the tumor. The proposed mechanisms of action include dielectrophoresis, disruption of the mitotic spindle apparatus, impaired cellular proliferation, and interference in various intracellular pathways, including regulation of DNA repair, autophagy, apoptosis, and immunomodulation (22–30).

Several studies have simulated the distribution of TTFields in the cranium (31, 32), chest (33), and abdomen (34, 35). Notably, bone tissue contained within these models consistently retained an electrical field intensity greater than 1 V/cm, which is considered the minimal threshold for in vivo efficacy (25, 36). In this context, we designed a series of experiments to evaluate the use of TTFields for radiation-refractory spinal metastases, including: (a) computational modeling of TTFields distribution in the human torso to simulate the anatomical changes related to surgery for spinal metastasis, (b) in vivo studies evaluating the antiproliferative effect of TTFields in 3D cultures of lung adenocarcinoma and osteosarcoma cell lines growing inside human bone scaffolds, and (c) in vivo experiments using an orthotopic murine model of spinal metastases to investigate the impact of TTFields on cellular proliferation, radiographic progression, histologic changes, and neurologic outcomes. Taken together, these results are the first to our knowledge to provide substantive evidence for TTFields as a viable treatment option for patients with spinal metastases. Using these data as a foundation, work is underway for the clinical investigation of TTFields in the treatment of osseous tumors.

## Results

### Computational model of TTFields distribution in human torso with a simulated laminectomy and spinal stabilization.

In order to model the application of TTFields in the setting of spinal metastases, we performed a simulation study of a typical surgical approach to address recurrent metastatic spinal cord compression. In this scenario, we modeled a laminectomy, resected the pedicles and posterior third of the vertebral body, added an expected postoperative seroma accumulating around the thecal sac, and applied a titanium screw/rod construct spanning 2 levels above and below the tumor resection. We simulated the transcutaneous delivery of 150 kHz TTFields and the same transducer arrays clinically approved for the treatment of malignant pleural mesothelioma.

**Figure 1:**
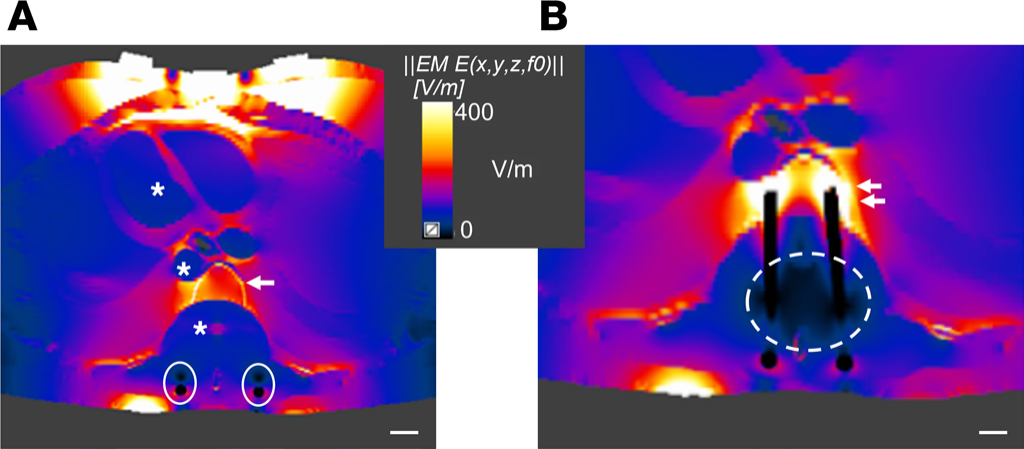
Computational model of TTFields distribution in the human torso with a simulated laminectomy and spinal stabilization for management of spinal tumor. (**A**) Computational model in axial view, centered at a simulated laminectomy and resection of bilateral pedicles and posterior vertebral body for decompression of the spinal cord. Note that the value of the TTFields intensity was consistently low (<1 V/cm) in regions containing bodily fluids such as chambers of the heart, great vessels, seroma of the resection cavity, and thecal sac (*). The titanium rods are conductive and shunted the electrical field in the surrounding seroma, creating a second adjacent area of minimal field deposition (white closed circles). The residual vertebral body (arrow) retained a higher TTFields intensity (2–3 V/cm), as it has a lower conductivity than that of the surrounding tissue layers. (**B**) Computational model in axial view at the level adjacent to the tumor resection. At this level, titanium screws were incorporated into the vertebral body and the conductive nature of the metal shunted the electrical field from the seroma and adjacent tissue, thus creating a zone around the implants that have attenuated TTFields intensity (<1 V/cm, dashed circle). However, as the conductive hardware joined the nonconductive bone, it allowed for a greater retention and boost of TTFields intensity (4 V/cm) deposition in the bone (double arrow). Scale bar: 1 cm.

The results predicted a low intensity (<1 V/cm) TTFields distribution in the seroma of the resection cavity, thecal sac, heart, and great vessels (Figure 1A). This is related to the high electrical conductivity of body fluids. As expected, the deposition of TTFields inside and around the spinal hardware was 0 V/cm, as these are considered nearly pure conductors. The residual vertebral bone of the resected tumor demonstrated substantial accumulation of TTFields intensity as a result of its lower conductivity compared with surrounding structures. This unique feature of the vertebral body facilitated enhanced TTFields intensities, with a range of 2–3 V/cm. Furthermore, the adjacent levels with titanium instrumentation received a boosted intensity to 4 V/cm as the electrical field was shunted toward the bone (Figure 1B).

### Effects of TTFields in vitro.

**Figure 2:**
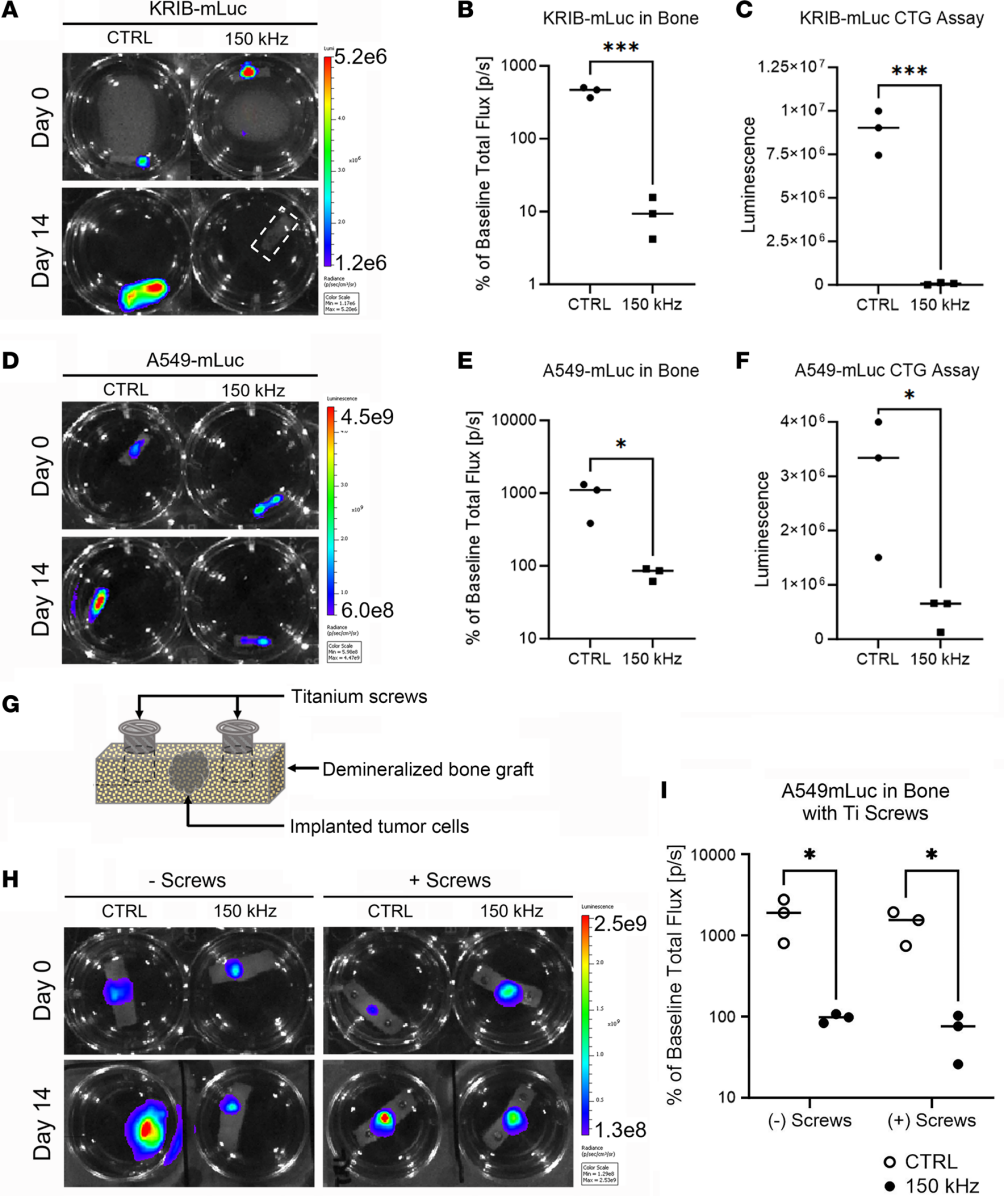
Effect of TTFields on tumor cell growth in 3D bone graft tumor model, with or without titanium screws in the treatment field. KRIB-mLuc cells (**A**–**C**) and A549-mLuc cells (**D**–**F**) were 3D cultured in demineralized bone matrix scaffolds and exposed to 150 kHz TTFields for 14 days. Tumor cell growth was monitored by BLI on days 0 and 14 (**A** and **D**). Control groups had significantly higher BLI signal on day 14 relative to baseline (day 0) compared with TTFields-exposed cultures (**B** and **E**). Control groups also contained significantly more viable cells (CellTiter-Glo [CTG] assay) on day 14 than the TTFields-exposed groups (**C** and **F**). A549-mLuc cells were cultured in demineralized bone matrix scaffolds with or without titanium screws to model the pedicle screws implanted in patients (**G**) and exposed to 150 kHz TTFields or control for 14 days. Tumor cell growth was monitored by BLI on days 0 and 14 (**H**). Control groups had significantly higher BLI signal on day 14 than TTFields-exposed groups, relative to respective baseline values. Additionally, the close proximity of titanium screws had no effect on the antiproliferative effects of TTFields exposure (**I**). Values represent mean ± SEM from independent experiments (*n* = 3). *P < 0.05, ***P < 0.0005 by unpaired, 2-tailed *t* test.

With modeling data to support enhanced TTFields deposition in bone, we next attempted to evaluate the ability of TTFields to inhibit growth of tumor cells within bone matrix, which simulates tumor formation inside the vertebral body. The experiments were performed using KRIB-mCherry-luciferase (KRIB-mLuc) osteosarcoma and A549-mCherry-luciferase (A549-mLuc) lung adenocarcinoma cell lines, which display strong bone tropism and form aggressive tumors in animal models (37). Initial in vitro cytotoxicity screens for each cell line were performed as described in Kirson et al., with varying TTField frequencies ranging from 50–250 kHz, and we identified 150 kHz as the optimal frequency for both KRIB-mLuc and A549-mLuc (36). Tumor cells were subsequently seeded in bone grafts, and after 96 hours, bioluminescence imaging (BLI) was used to confirm engraftment and establish baseline levels for each sample (Figure 2, A and D). The tumor-containing bone grafts were exposed to 150 kHz TTFields, while control samples were not exposed. Compared with control samples, application of TTFields resulted in significant growth inhibition after 14 days in bone graft models for each cell line. KRIB-mLuc BLI signal decreased to 10% of the baseline measurement, while control samples displayed a 4.48-fold increase over baseline levels (*P* = 0.0005; Figure 2B). Similarly, TTFields exposure to A549-mLuc bone grafts decreased BLI signal to 80% of baseline readings versus a 9.36-fold increase over baseline in control samples (*P* = 0.0386; Figure 2E). Consistent with this result, viable cell number at study endpoint was 125-fold higher with KRIB-mLuc cells (*P* = 0.0003) and 6-fold higher with A549-mLuc cells (*P* = 0.0325) under control versus TTFields exposure conditions (Figure 2, C and F). Taken together, these results reflect marked TTFields-mediated inhibition of tumor cell growth in 3D culture bone grafts.

In the treatment of spinal tumors, it is often necessary to stabilize the spine with titanium screws. We thus explored whether the metallic properties of this instrumentation could potentially disrupt the electrical field delivery in the setting of TTFields exposure. A549-mLuc cells were seeded in demineralized bone grafts without or with two 6-mm titanium mandible screws (Figure 2G) placed in the edges of the bone scaffold to simulate a tumor growing inside a vertebral body in the presence of titanium pedicle screws. As a control, A549-mLuc bone grafts without titanium screws were used. After 96 hours in culture, BLI was used to confirm engraftment and establish baseline levels for each sample (Figure 2H). The bone grafts were then exposed to 150 kHz TTFields or mock treated. We observed consistent growth inhibition due to TTFields after 14 days under both conditions (with or without screws) compared with controls. Bioluminescence signal from both TTFields-exposed conditions decreased to 75% of baseline, whereas for both control conditions, it increased 12.25-fold over baseline (with screws: *P* = 0.037375, without screws: *P* = 0.037993; Figure 2I). These results suggest that the inhibitory effect of TTFields on tumor growth was not disrupted by the presence of titanium screws in the treatment field.

### Effects of TTFields in vivo.

**Figure 3:**
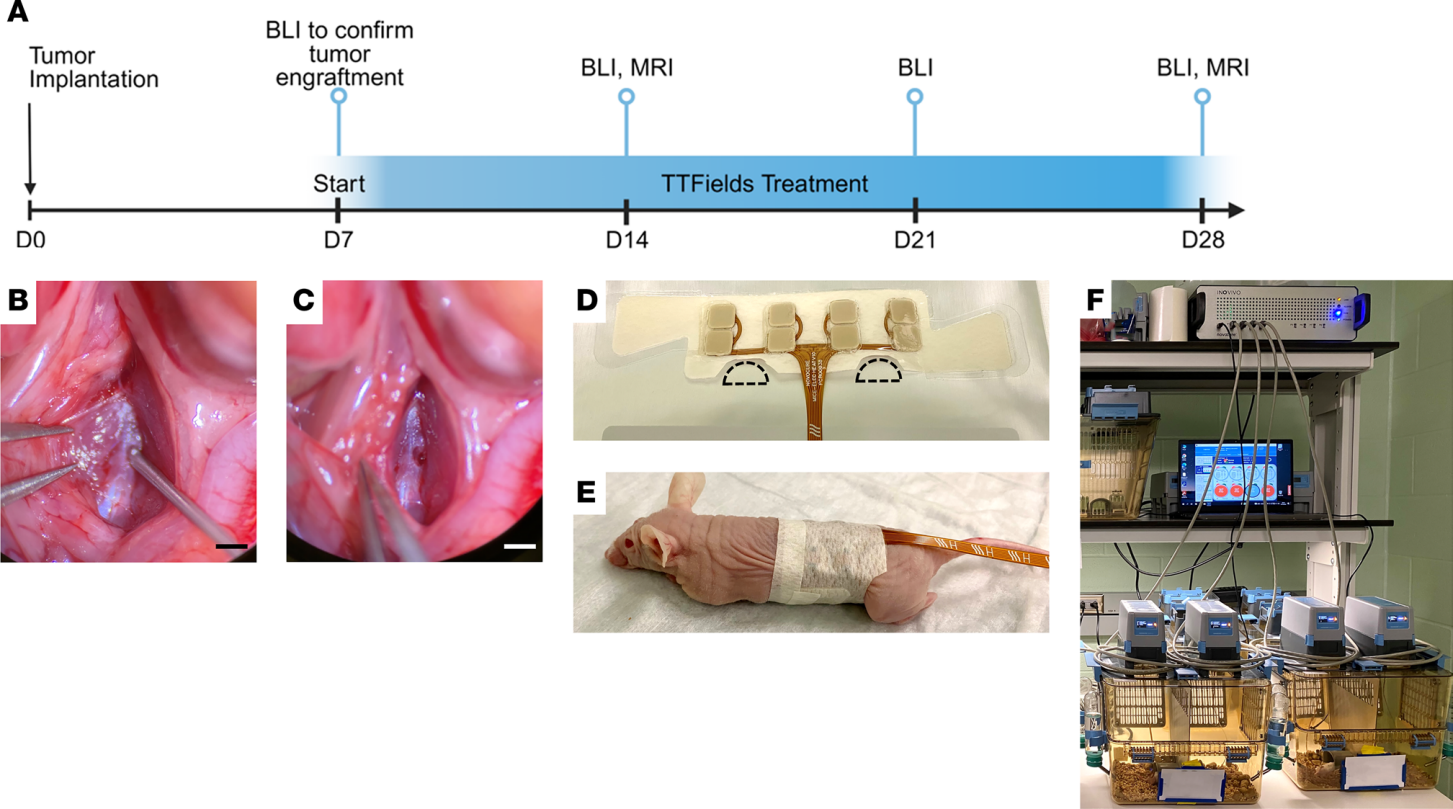
In vivo tumor implantation and TTFields or sham control (heat) delivery. (**A**) Timeline schematic for tumor implantation, TTFields or sham heat exposure, and imaging for in vivo experiment. KRIB-mLuc tumor cells were implanted via 26-gauge Hamilton syringe (**B**) through a burr hole into the mouse vertebral body (**C**). Scale bar: 1 mm. Inovivo transducer arrays (**D**) were trimmed (dashed lines) to allow for placement over the lumbar spine without restricting movement of mouse hind limbs (**E**). Mice were subsequently placed in the inovivo system consisting of a cage, field generator, cage connections, and control/monitoring software (**F**).

**Figure 4:**
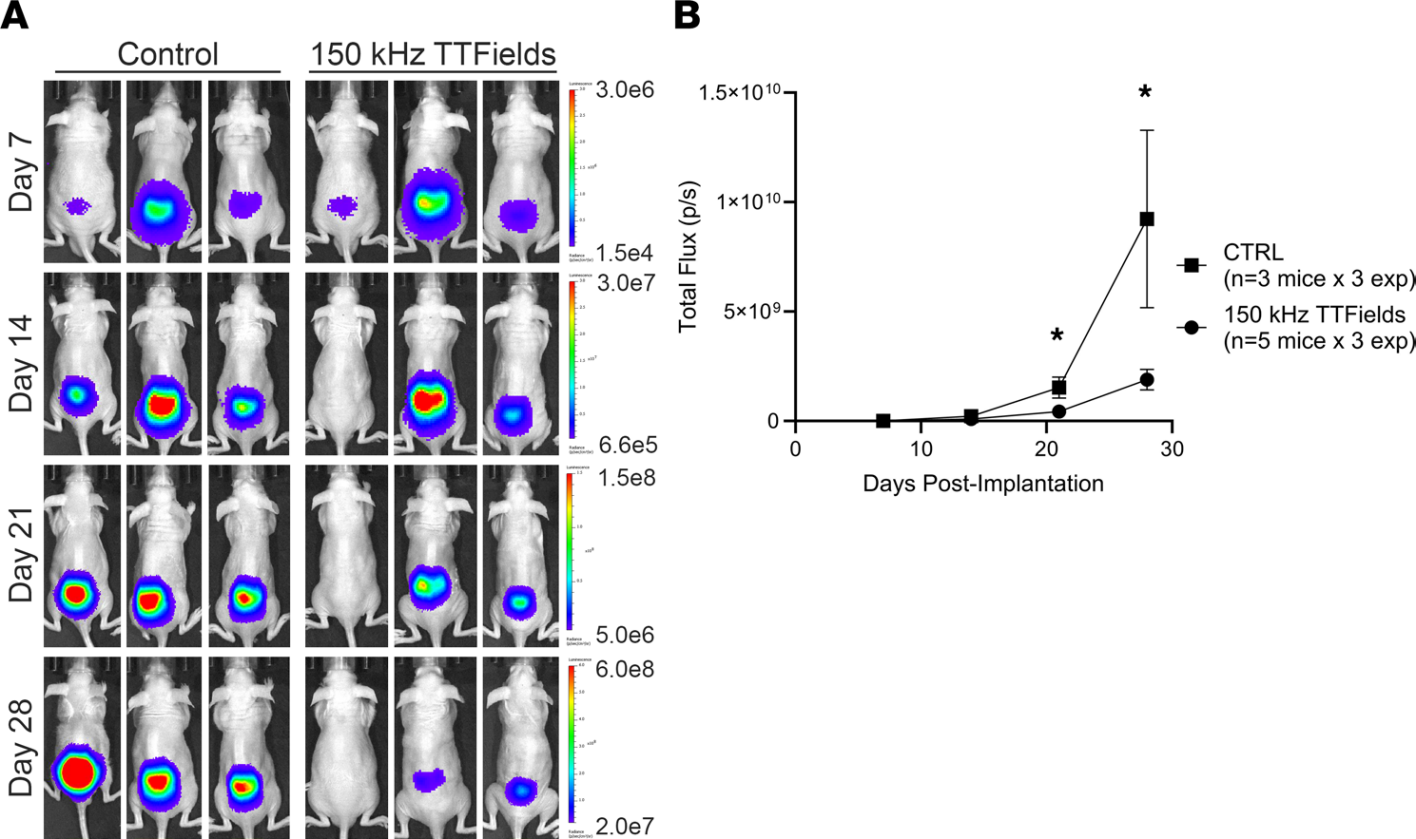
The effect of 150 kHz TTFields versus sham control (heat) administration on tumor cell growth in a murine orthotopic model of spinal metastasis. KRIB-mLuc orthotopic xenograft tumors in nude mice were treated with sham control (heat) or 150 kHz TTFields and tumor growth was monitored by weekly BLI. Representative bioluminescence images were taken on postoperative days 7, 14, 21, and 28 (**A**). BLI signal was significantly higher on day 21 (*P* = 0.0226) and day 28 (*P* = 0.0428) in control versus TTFields-exposed mice (**B**). Values represent mean ± SEM (*n* = 5 treated animals, 3 control animals) from 3 independent experiments, with statistical comparisons by unpaired, 2-tailed *t* test at each time point per experiment. *P < 0.05.

Our next aim was to assess the ability of TTFields to inhibit the in vivo growth of intraosseous spinal tumors and associated spinal cord compression leading to neurologic decline using an orthotopic xenograft mouse model. Due to the optimal in vivo growth kinetics of KRIB-mLuc osteosarcoma cells for subsequent inovivo (Novocure Ltd.) treatment, KRIB-mLuc cells were implanted orthotopically into the vertebral body of the lumbar spine in athymic nude mice (Figure 3, A–C). Tumor engraftment was confirmed by BLI on postoperative day 7, prior to initiation of treatment. Mice were randomly assigned to sham control (heat) or 150 kHz TTFields exposure groups, delivered via flexible torso transducer arrays (Figure 3, D–F). Weekly BLI of the entire cohort and MRI of select mice from each group were used to monitor tumor growth (Figure 4A). Control mice demonstrated a 3.5-fold higher mean BLI signal than mice receiving 150 kHz TTFields on day 14 (*P* = 0.0226), and the same comparison was 5-fold higher on day 21 (*P* = 0.0428; Figure 4B).

**Figure 5:**
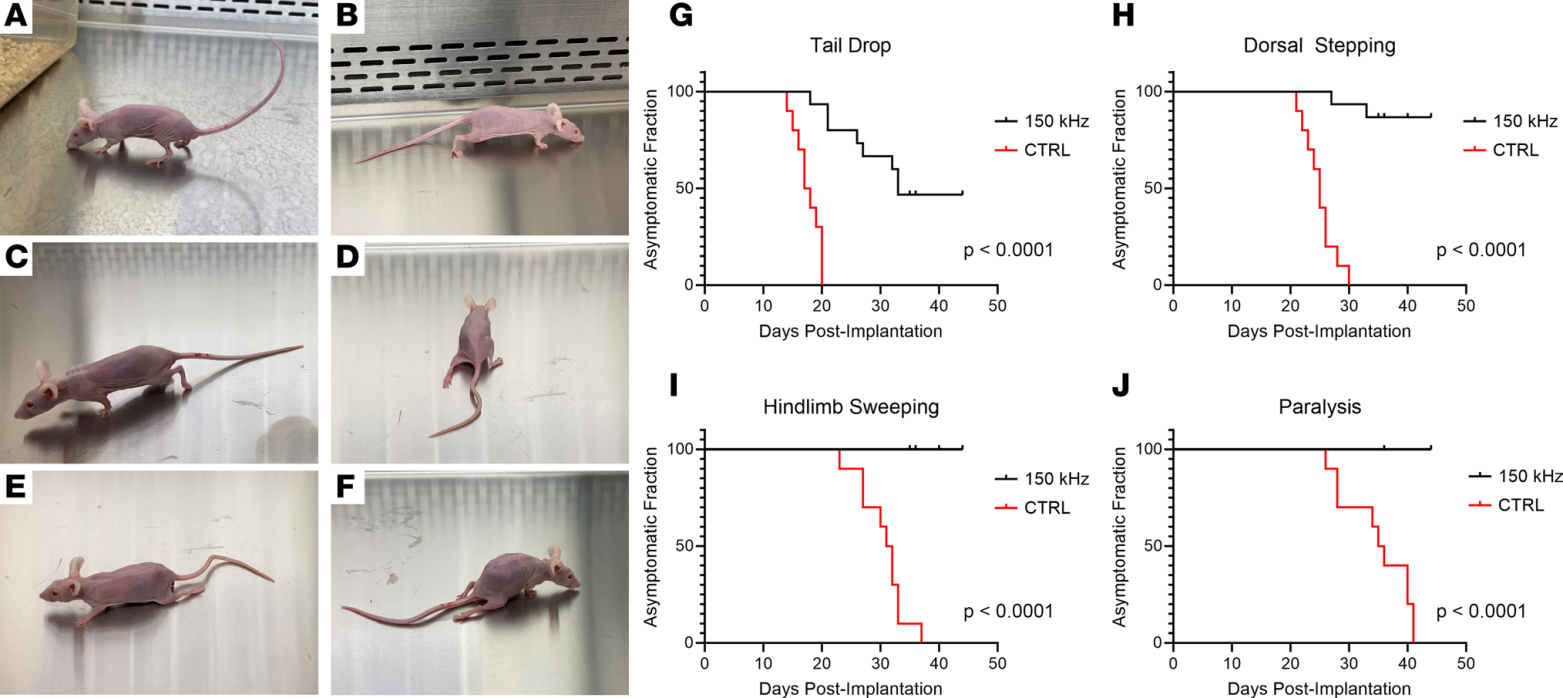
Effects of 150 kHz TTFields versus control (heat) on neurologic functional status milestones in mice bearing spinal tumors. Mice demonstrating normal posture (**A**) and progressive paralysis milestones of tail drop (**B**), dorsal stepping (**C**), hind limb sweeping (**D** and **E**), and hind limb paralysis (**F**). Control mice displayed a median time-to-tail drop of 17 postoperative days and all others experienced tail drop by 20 days after implantation, whereas 53% of TTFields-exposed mice displayed symptoms on day 33 and the other 47% remained symptom free (**G**). Control mice had a median time-to–dorsal stepping of 25 days, while 2 of 15 TTFields-exposed mice displayed dorsal stepping over the observation period (on days 27 and 33) (**H**). Control mice had median time-to–hind limb sweeping at 31.5 days and median time-to–bilateral hind limb paralysis at 35.5 days; in contrast, no TTFields-exposed mice progressed to either of these milestones by the end of the observation period (**I** and **J**). Statistical comparison of asymptomatic fractions performed using log-rank (Mantel-Cox) test (*n* = 5 treated animals and 3 control animals, per experiment).

We evaluated the neurologic functional status between TTFields-exposed and control animals. Previous studies using this orthotopic tumor model have shown milestones of neurologic symptoms corresponding to the degree of spinal cord compression, beginning with tail drop and progressing through dorsal stepping, hind limb sweeping, and hind limb paralysis (Figure 5, A–F) (38, 39). Mice were observed daily for symptoms of spinal cord compression and the date of occurrence of these milestones was recorded. In all experiments, TTFields-exposed mice showed delay in development of symptoms or no symptoms at all. In control animals, the median time to the occurrence of tail drop was 17 days after tumor implantation, compared with 33 days in the TTFields group, with 7 of 15 TTFields-exposed animals remaining symptom free (*P* < 0.0001; Figure 5G). Additionally, the median time to dorsal stepping in the control group was 25 days, while only 2 TTFields-exposed mice displayed dorsal stepping at the conclusion of the study (day 27 and day 33, respectively, *P* < 0.0001) (Figure 5H). Lastly, control mice displayed hind limb sweeping at a median time of 31.5 days and bilateral hind limb paralysis by a median time of 35.5 days after implantation. In contrast, no TTFields-treated animals progressed to either of these milestones by the end of the study (*P* < 0.0001; Figure 5, I and J).

**Figure 6:**
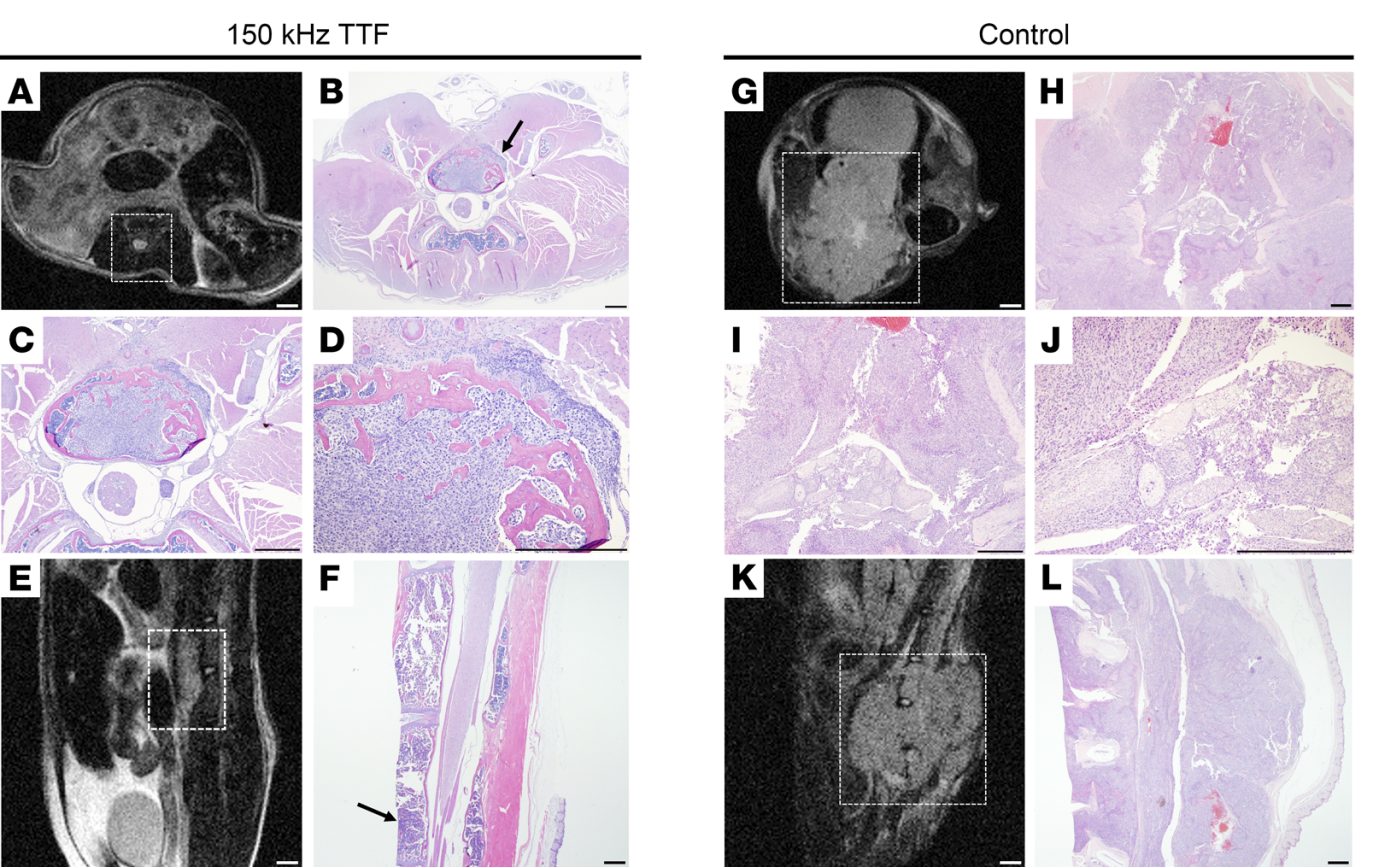
MRI and histological comparison of tumors exposed to 150 kHz TTFields versus sham control (heat). Representative T2-weighted MR images from axial (**A** and **G**) and sagittal (**E** and **K**) orientations shown at the plane of tumor implantation in TTFields-exposed mice (**A** and **E**) versus control mice (**G** and **K**). Histological analysis of tissues collected from the same mice, displaying axial sections at the same plane as corresponding MR images at ×10.25 magnification (**B** and **H**), ×40 magnification (**C** and **I**), and ×100 magnification (**D** and **J**), as well as sagittal sections at ×10.25 magnification (**F** and **L**). Black arrows indicate tumor implantation site. MRI scale bars: 1 mm. H&E scale bars: 500 μm.

In addition to surveillance of neurologic function in response to TTFields exposure, longitudinal MRI was performed on days 14 and 22 to corroborate BLI observations. To minimize stress of extended anesthesia and handling, MRI was performed on 1–2 mice/group in each experiment. In all mice imaged, approximate tumor volume and location were consistent between MRI and BLI results. MRI revealed that tumors in all imaged control mice invaded multiple vertebral levels, paraspinal musculature, and caused substantial compression of the thecal sac and spinal cord, while tumors in the 8 imaged TTFields-exposed mice (out of 15 total) remained confined to the implanted vertebral body (Figure 6).

For each experiment, T2-weighted MRI sequences were obtained on day 21 to visually confirm tumor cell implantation and assess the degree of paraspinal/epidural tumor cell growth. Mice were subsequently euthanized at the time of paralysis in the control-treated group or postoperative day 40, per protocol, in the TTFields-treated group, as none of these mice displayed paralysis. Tissues were then collected for histological analysis. Histologic findings were consistent with BLI, MRI observations, and neurologic assessments, with TTFields-exposed mice having reduced intravertebral tumor size, limited growth into the epidural space with reduced spinal cord compression, and limited invasion into paraspinal musculature compared with control mice (Figure 6). In contrast, tissue from control mice demonstrated large osteolytic tumors with multiple levels of vertebral body invasion, severe spinal cord compression, and extensive invasion into adjacent musculature. These radiographic and histologic observations corresponded with the severe neurologic decline seen in control-treated mice and is consistent with past observations using this orthotopic tumor model (Figure 6) (38, 39).

## Discussion

While the applicability of TTFields has been explored with both preclinical experiments and simulation studies across various tumors (31, 32, 40–43), the greatest clinical advances have been in glioblastoma (20, 21) and malignant pleural mesothelioma (19) for which use of TTFields has been FDA approved. With those encouraging results, further studies are underway investigating TTFields in other solid organ carcinomas (e.g., pulmonary, hepatic, ovarian, pancreatic, and gastric). As part of these efforts, several computational models have simulated TTFields distribution for the treatment of primary tumors in visceral organs. TTFields are delivered via cutaneous transducer arrays and the electrical field must penetrate the different tissue layers to exert its antimitotic effect on the cancer cells growing inside the target organ. The electrical conductivity and dielectric properties of each tissue layer overlying the tumor influence the distribution and intensity of TTFields reaching the tumor. Due to the unique physical properties of bone and its low conductivity, we hypothesized that spinal metastases would respond well to the antimitotic features of TTFields administration.

The conductivity of tissue is inversely proportional to the retention of the electrical field (40). In order to apply this concept to spinal metastases, we leveraged the understanding of TTFields distribution for the treatment of glioblastoma, in which the electrical properties of the tissue layers overlying the parenchymal tumor influence the intensity of TTFields at the target tumor. First, the skull, which has a lower electrical conductivity than the adjacent layers such as subcutaneous tissue and dura, attenuates the voltage reaching the deeper tissue. Simulation studies demonstrate increased intensity of electrical fields inside brain tumors when strategic bone resections are performed to mitigate against the attenuation of TTFields intensity caused by the skull (31, 44). Second, the brain is surrounded by cerebrospinal fluid, which is more conductive than the underlying gray matter, thereby creating a shunt effect that decreases the deposition of TTFields intensity into the brain parenchyma and intraparenchymal tumor (40).

We believe the electrical properties of bone and anatomical changes following surgery for spinal metastases provide a unique collateral benefit for the application of TTFields in the postoperative setting. Our computational model indicated that the removal of bone in the epidural space (i.e., laminectomy and pediculectomy), in addition to the presence of a postoperative seroma, decreased the impedance of the tissue layers, facilitating the penetration of the electrical field up to the level of the epidural space and vertebral body. These studies are consistent with the work of Bomzon et al., which explored electrical field distribution and associated intensity for the treatment of lung malignancies (33). Consistent with our results, the electrical field intensity exceeded 1 V/cm in the intact vertebral body. Similarly, Lok et al. examined the physical properties of various anatomic structures and noted that cancellous bone, cortical bone, and the intact spine were notable for their high physical density and low electrical conductivity (45). Taken collectively, these results suggest that bone of the spinal column retains substantial electrical charge and associated TTFields intensity.

Additionally, the presence of titanium instrumentation above and below the surgical decompression created a shunt effect, increasing the electrical field intensity within the intact, adjacent vertebral levels. We believe this feature could improve the therapeutic efficacy of TTFields in the setting of recurrent spinal metastasis, as higher field intensity in the adjacent levels could prevent hematogenic seeding or direct tumor invasion via Batson plexus, as we have observed in our in vivo experiments (Figure 6, A–F). This is the first description to our knowledge of a potential benefit of metallic implants shunting electrical field intensity in a tumor-directed manner in the setting of TTFields therapy. Improved modeling and inclusion of different materials like carbon fiber implants need to be investigated in future studies.

We provide the first description to our knowledge of laboratory experiments evaluating the influence of TTFields in the proliferation of cancer cells inside bone. Most in vitro investigations of TTFields use monolayer cell culture and a shorter time duration of exposure (18). Our approach used a 3D cell culture model of tumor cells inside a matrix of demineralized bone kept in culture for 14 days (Figure 2). This experimental setup allowed us to evaluate the effects of long-term exposure to TTFields in the unique osseous microenvironment and supports the results of our computational model that predicted therapeutic levels (>1 V/cm) within the vertebral body after surgical resection of a spinal metastasis. We demonstrated that tumor-associated bioluminescence signal and cell viability are significantly reduced by application of TTFields (Figure 2, H and I) in the presence and absence of titanium pins. This is particularly notable given the previous finding that 200 kHz TTFields increases the permeability of cancer cell membranes, thereby permitting enhanced entry of luciferin into TTFields-exposed cancer cells, resulting in higher bioluminescent signal (46). Although there may be a difference in the cancer cell–permeabilizing effects of 200 kHz versus 150 kHz TTFields, and accounting for the difference in cell membrane structure in human glioblastoma versus osteosarcoma and lung adenocarcinoma cells, the large magnitude of difference in BLI signal between the TTFields-exposed and control conditions in the current study suggests a TTFields-inhibitory effect on cancer cell proliferation in the investigated cancer cell lines. Furthermore, the BLI results in this study were confirmed with an independent ATP-based quantitative read-out, CellTiter-Glo.

We transitioned to an orthotopic murine model of spinal metastasis to evaluate whether the antiproliferative effects observed in vitro could be recapitulated in vivo. Our orthotopic model facilitated identification of progressive neurologic deficits associated with increased tumor burden. Simulation studies of TTFields distribution in a rat model demonstrated that the spine retained approximately 3 V/cm when TTFields were applied using transcutaneous arrays around the thoracic region (47). Building on these initial modeling studies, we demonstrate for the first time to our knowledge a correlation between computational model predictions and neurologic functional benefit in vivo.

Mice exposed to TTFields had significant delays in developing neurologic deficits when compared with controls (Figure 5), and this was corroborated with BLI (Figure 4) and MRI (Figure 6). We demonstrated that using 150 kHz was associated with a 5-fold reduction in the BLI signal in the TTFields group compared with control (Figure 4). While our study was designed to evaluate the effects of continuous TTFields treatment up to 30 days, the presence of the transducer arrays was irritating to the mice and resulted in animals attempting to disconnect the arrays. As a post hoc analysis of the EF-14 clinical trial demonstrated (20), the survival-prolonging benefit of TTFields positively correlates with duration of “on time” exposure (48). In this context, we confined our study to mice that received more than 18 hours of treatment per day, which was the cutoff used in the EF-14 trial (20) and part of the FDA label for patients with newly diagnosed glioblastoma. Future studies are required to directly link the duration of TTFields exposure to therapeutic efficacy in spinal metastasis models.

MR images obtained at 14 and 21 days after treatment initiation demonstrated marked reductions in tumor size and limited spread to adjacent vertebral bodies in the mice treated with TTFields (Figure 6). This is a critical observation for the management of recurrent spinal metastasis, as the epidural spread and involvement of adjacent vertebral bodies often requires an increase in the magnitude of surgical intervention and elevates the risk of complications in a patient population that is already typically frail. Lastly, histological analysis of the tissue samples confirmed that TTFields exposure was associated with marked suppression of tumor growth (Figure 6).

Our in vivo experiment confirmed that TTFields are able to penetrate the bone microenvironment, which included both cortical and trabecular bone with intact calcium contents, as opposed to our in vitro studies that used demineralized bone. It is possible that TTFields can exert a therapeutic effect not only in the postoperative setting to prevent tumor recurrences from the residual bone toward the epidural space, but also in spine levels not included in the laminectomy site and even bone sites not related to the spine, such as the long bones or skull base.

This is the first study to our knowledge to consider bone as a target for treatment with TTFields, converting a previously supposed limitation into a targeted therapeutic asset. A limitation of this study is the inability to directly measure the electrical field intensity. As a result, computational models quantifying electrical field intensity are indirectly validated by the biologic effect observed in cell cultures (22–25) or in the survival benefit observed in clinical trials (19–21). Our results validate prior rat simulation studies (47) predicting electrical field intensities of up to 3 V/cm in the dorsal region of murine models, which includes the spine. We demonstrated that application of TTFields in this predicted intensity range correlated with suppression of tumor growth inside the vertebral body, reduced tumor dissemination to adjacent levels, and prevented spinal cord compression and neurologic decline.

In conclusion, this study demonstrates that TTFields can be used to target osseous tumors in vivo. Whereas the 150 kHz TTFields frequency was used in this proof-of-concept study, future considerations will include the in vitro determination of an optimal cell-killing frequency in cells derived from a patient’s resected metastatic spinal tumor, to create a personalized TTFields therapy approach. As the survival of cancer patients improves and the incidence of spinal metastases increases, maintenance of both quality of life and functional status is a significant goal. These results support a clinical trial to evaluate the use of transcutaneous TTFields for the management of radiation-refractory spinal metastasis. Additionally, the combination of TTFields with concomitant modalities including immunotherapy, targeted therapies, and adjuvant radiation therapy could be evaluated. Further studies are needed to evaluate whether the changes in tissue conductivity and permittivity related to surgery, and the use of metallic implants, can enhance the therapeutic effects of TTFields in bone tumors.

## Methods

### Sex as a biological variable.

Male and female nude mice were used for in vivo experiments and mixed randomly between treatment and control groups.

### Computational model of TTFields distribution in the human torso with simulated laminectomy and pedicle screw placement.

Simulations were performed using the Sim4Life v6.1 software package (ZMT Zürich MedTech AG). The ELLA computational phantom of a healthy 26-year-old female (ZMT Zürich MedTech AG) was used for this study. The permittivity and conductivity were assigned to the tissues of the phantom based on the model described by Gabriel et al. (49), which was built into the software material database. Tissue conductivity was modified based on Hershkovich et al. (34). Electrical field distributions were calculated using the Sim4Life’s Ohmic-quasistatic low-frequency finite element solver. Bone covering the spinal cord (spinous process, lamina, pedicles, and posterior third of vertebral body) was removed from the model and replaced with conductivity and permittivity values similar to serum to reproduce the typical fluid accumulation observed in the initial postoperative stages. Linear 5-mm-thick rods with conductivity comparable to titanium were added at the vertebral bodies 2 levels above and below the simulated resection to replicate the standard spinal hardware construct. TTFields intensity was denoted throughout the simulated resection cavity.

### Cell lines and maintenance.

The human KRIB osteosarcoma cell line (a gift of Valerae O. Lewis, MD Anderson Cancer Center) and human A549 lung adenocarcinoma cell line (ATCC) were transduced with an mCherry-Luciferase dual reporter lentiviral vector. KRIB-mLuc and A549-mLuc cell lines were maintained in DMEM/F12 (Corning Life Sciences) with 10% fetal bovine serum, penicillin-streptomycin (50 U/mL/50 μg/mL) (Gibco), and GlutaMAX (Gibco) in a humidified incubator with 5% CO_2_/95% room air at 37°C.

### Cell culture.

A total of 2.5 × 10^4^ KRIB-mLuc osteosarcoma cells or A549-mLuc lung adenocarcinoma cells were seeded in the center of a 12 × 5 mm demineralized human cancellous bone graft (MTF Biologics) with or without 6-mm titanium mandible screws (Synthes) placed in the edges of the bone scaffold. Cultures were maintained in complete growth medium in a humidified incubator with 5% CO_2_/95% room air at 37°C. After seeding on the bone scaffold, cultures were incubated for 96 hours and imaged by BLI to confirm engraftment prior to exposure with TTFields using the inovitro system (Novocure Ltd.).

### BLI and quantification.

Bone grafts and mice were imaged using the IVIS Lumina XR System (Caliper Life Sciences) and analyzed using Living Image Software (Caliper Life Sciences). For in vitro studies, 750 μg (50 μL of 15 mg/mL) D-luciferin (GoldBio) was added directly to 3 mL culture media 1 minute prior to BLI acquisition. For in vivo experiments, mice were injected subcutaneously with 3 mg (0.2 mL of 15 mg/mL) D-luciferin 15 minutes prior to BLI acquisition. Bioluminescence color images were overlaid on gray-scale photographic images of the animals to allow for localization of the light source using the Living Image software overlay (Caliper Life Sciences). Circular regions of interest (ROIs) were manually selected with fixed dimensions (1.786 cm diameter), and signal intensity was expressed as total flux (photons/second). Image scales were standardized to enable visual comparisons across samples and between groups.

### Application of TTFields in the 3D culture.

After tumor engraftment was confirmed with BLI, the bone grafts seeded with cells were transferred to the inovitro system ceramic dishes, which contain 2 perpendicular pairs of transducer arrays within their walls (50). The dishes were connected to the TTFields generators through specialized wired baseplates, and TTFields was applied at 150 kHz. Incubator temperature was set to 26°C (observed range 24°C–28°C), with a target temperature of 37°C for culture dishes during TTFields exposure, to produce an expected field intensity of 1.04 V/cm inside each dish (50). Control (no TTFields) dishes were maintained in a humidified incubator with 5% CO_2_/95% room air at 37°C with no TTFields exposure. Temperature and connection of each dish were monitored continuously by the inovitro system software, and culture media were replaced daily during the 14-day period. Experiments were paused for less than 30 minutes for BLI at each imaging time point, and 3D cultures were transferred to 12-well plates (Corning Life Sciences) for BLI. After imaging, cultures were transferred back to ceramic dishes containing fresh media and experiments resumed. BLI images were obtained on days 0, 7, and 14. Each experiment consisted of *n* = 3 TTFields exposure and *n* = 3 control samples, and 3 independent such experiments were performed.

### Cell viability assays following application of TTFields in 3D cultures.

A total of 2.5 × 10^4^ KRIB-mLuc or A549-mLuc cells were seeded in demineralized bone grafts, cultured for 96 hours, imaged by BLI to confirm engraftment, and subsequently treated with or without 150 kHz TTFields using the inovitro system. Immediately following each 14-day experiment, bone grafts were transferred to opaque-walled 24-well plates and CellTiter-Glo 2.0 luminescent cell viability assay (Promega) was performed according to the manufacturer’s instructions. Following incubation of CellTiter-Glo reagents, luminescence measurements and data quantification were performed using a Clariostar microplate reader with MARS data analysis software (BMG Labtech).

### Development of the orthotopic murine model of spinal metastasis.

The surgical implantation of tumors in the lumbar spine via a transperitoneal approach has been previously described (38). Briefly, athymic nude mice (male and female, age 6–8 weeks, weighing 25–30 g) were anesthetized with 2% isoflurane, placed in a supine position under a dissecting microscope, and 30 μL (0.5 mg/mL) buprenorphine extended-release analgesic was administered by subcutaneous injection. Isoflurane anesthesia administered by nose cone was maintained for the duration of the procedure. A midline incision in the abdomen was performed, the small and large bowels were retracted with fishhooks, and the aorta and vena cava identified. The vascular bundle was dissected at the level of the inferior pole of the left kidney and the underlying psoas muscle retracted laterally to expose the anterior surface of the vertebral bodies (approximately L2-L3 level). A hand drill was used to create a small hole in the anterior cortex of the exposed vertebral body and 2.5 × 10^4^ KRIB-mLuc cells suspended in 3 μL Cultrex Basement Membrane Extract, Type 3 (R&D Systems) was injected into the hole using a 26-gauge Hamilton syringe. The peritoneal cavity was closed by layers. The animals were allowed to recover and transferred to standard cages. BLI was performed 7 days after tumor implantation to confirm engraftment prior to TTFields administration.

### Application of TTFields for treatment of spinal metastasis.

After tumor engraftment was confirmed in each animal via BLI, 2 groups of *n* = 5 animals each were randomized to receive control (heat) or 150 kHz TTFields exposure, delivered using the inovivo system. The procedures for handling, array application, and housing of animals were performed as described by Blatt et al. (47), with the slight modification of trimming the cloth backing of the arrays to allow for hind limb movement, as we applied the arrays on the abdomen overlying the bioluminescence signal of the developing spinal tumor. The optimal array contact with the animal’s skin was indicated by resistance (185–400 Ω) and was monitored continuously throughout the treatment period. Arrays were replaced every 2–3 days to maintain optimal contact and resistance, or as needed when damaged by the animals. Treatment was applied continuously for 3 weeks and only paused for weekly imaging (<3 hours) or daily neurologic analysis (<10 minutes) of the animals. Heat or TTFields exposure was maintained for a minimum of 126 hours per week (18 hours per day). Animals that received less than the weekly minimum exposure duration were excluded from analysis. Mice in the control group were treated with sham heat electrodes of the same size, weight, and shape, which resulted in only superficial heating similar to that caused by the TTFields electrodes. Each in vivo experiment was repeated 3 times, independently.

### Neurologic analysis.

Animals were observed daily for 5 minutes and the day of occurrence of (a) tail dragging, (b) dorsal stepping, (c) hind limb dragging, and (d) paralysis of one or both hind limbs were recorded, where day 0 was implantation of the tumor into the spine. The mean number of days ± standard error of the mean (SEM) for reaching each of the 4 milestones was calculated. Animals were euthanized when bilateral hind limb paralysis occurred in accordance with institutional guidelines.

### MRI images.

For all MRI experiments, animals were anesthetized with 2% isoflurane. Imaging was performed on a 7-T small animal MRI scanner (Biospec, Bruker Biospin MRI, Inc.) using transmit/receive volume coils with 35 mm inner diameter developed by MD Anderson Cancer Center Small Animal Imaging Facility personnel. Heat and TTFields transducer arrays were removed prior to MRI acquisition. T2-weighted image sequences were obtained in coronal, axial, and sagittal orientations with 0.75 mm slice thickness. After confirmation of BLI signal in the spine 7 days after tumor implantation, animals were randomly selected from TTFields and control groups and longitudinally imaged on days 14 and 22 after tumor implantation.

### Histological analyses.

At study endpoints, animals were euthanized and perfused with 4 mL of 4% paraformaldehyde (Electron Microscopy Sciences). Lumbar spinal cord and adjacent tissues were collected and fixed and decalcified in EDF decalcifier (Statlab) and then processed for paraffin embedding and sectioning. Tissue sections (5 μm thick) were deparaffinized in xylene and rehydrated in a graded ethanol series, and then stained with H&E following standard methods and mounted with Cytoseal XYL (Thermo Fisher Scientific). H&E-stained sections were imaged using an Olympus BX53 microscope with Olympus CellSens software.

### Statistics.

Statistical analyses of in vitro and in vivo data were performed using GraphPad Prism 9 software. Baseline correction was performed to represent in vitro BLI values as a percentage of the values from the initial imaging time point. Shapiro-Wilk test for normality was used to confirm normal distribution of data. Statistical comparisons of in vitro data were performed using unpaired *t* tests of compiled mean values from 3 independent experiments (*n* = 3). Where multiple unpaired *t* tests were used, correction for multiple comparisons was performed using the Holm-Šídák method. Time-to-paralysis milestones are represented using asymptomatic fractions with statistical comparisons of treatment versus control groups by log-rank (Mantel-Cox) test. Animals removed from the study due to skin irritation preventing application of TTFields arrays were censored from subsequent statical analyses. Statistical significance was defined as a *P* value of 0.05 or less.

### Study approval.

All animal experiments were performed according to animal protocols approved by the Institutional Animal Care and Use Committee at MD Anderson Cancer Center.

### Data availability.

Individual values for data sets are available in the supplemental Supporting Data Values XLS file.

## Author contributions

DL, CT, and CAB designed the research studies. DL, RAADA, XW, AN, and CT conducted experiments. DL, RAADA, XW, and AN acquired data. DL, AN, CBP, CT, and CAB analyzed data. DL, CBP, QG, THB, RN, LR, JL, AG, DA, CT, and CAB wrote the manuscript.

## Supplementary Material

Supporting data values

## Version 1 03/21/2024

In-Press Preview

## Version 2 05/08/2024

Electronic publication
